# Overweight adolescents’ views on physical activity – experiences of participants in an internet-based intervention: a qualitative study

**DOI:** 10.1186/s12889-018-5324-x

**Published:** 2018-04-04

**Authors:** Turid Kristin Bigum Sundar, Knut Løndal, Per Lagerløv, Kari Galvin, Sølvi Helseth

**Affiliations:** 1 Department of General Practice, Institute of Health and Society, Faculty of Medicine, University of Oslo, Oslo, Norway; 2 Department of Nursing and Health Promotion, Faculty of Health Sciences, OsloMet - Oslo Metropolitan University, Oslo, Norway; 3 Department of Primary and Secondary Teacher Education, Faculty of Education and International Studies, OsloMet -Oslo Metropolitan University, Oslo, Norway; 4 VID Specialized University , Oslo, Norway; 5 Oslo, Norway

**Keywords:** Overweight and obese adolescents, Physical activity, Qualitative research interviews, Motivation, Health, Body image

## Abstract

**Background:**

Overweight and obese adolescents are reported to be less physically active than their peers. Research-based knowledge about their views may contribute to a better understanding of key factors that may foster or undermine motivation for physical activity, and provide knowledge for the future development of interventions. This paper explores experiences of physical activity among overweight adolescents, age 13-14 years, participants in Young & Active, a web-based controlled trial intervention to increase physical activity (ClinicalTrials.gov NCT01700309). The theoretical perspective is based on Self-Determination Theory.

**Methods:**

Two qualitative post-intervention research interviews, with a nine-month interval, were conducted with 21 adolescents, 15 girls and 6 boys to study short-term and long-term changes. The informants were recruited from a total of 84 participants from the Young & Active intervention group. Data were analyzed using qualitative content analysis.

**Results:**

The participants associated physical activity with organized sports and physical education classes at school, and as a means of promoting good health and attractive bodies. A majority of the adolescents said that they experienced their health as poorer than other youths, and expressed worries about their fitness and future health. Mastering a physical activity, being together with friends and having fun promoted motivation to perform sports. Not mastering an activity, or not knowing the others made them less motivated. None of the adolescents highlighted the importance of informal active living when asked about their understanding and experiences of physical activity. Consistency was found between the first and second interviews.

**Conclusion:**

This study adds to limited research on overweight and obese adolescents’ experiences of physical activity. The participants’ views reflect opinions in society about physical activity, and its importance for health. Viewing physical activity as conducted within organized sports makes it necessary to look into how these are organized, structured and led, and what can be done to support self-esteem, autonomous motivation and participation. The ability to choose among available, affordable and desirable physical activities, together with friends, may promote participation and maintenance.

## Background

Over the past three decades, the prevalence of overweight and obesity has increased in both adult and child populations worldwide, and is seen as a serious threat to public health [1]. A survey from Norway in 2015 among children aged 8 -9 years, showed that 17% of the girls and 13% of the boys were overweight, and that approximately 3% of the girls and 2.3% of the boys were obese [2]. Increased body mass index (BMI) at an early age is a major risk factor for developing non-communicable diseases. These conditions do not only cause long-term morbidity, but also premature mortality [3].

Studies show that the total amount of physical activity decreases from early childhood into adolescence [4, 5]. Fifteen-year-old adolescents are less active than nine-year-old children, and girls are less active than boys. Children from immigrant backgrounds or low education and low income families are less active than children from high income and high education families [6]. Overweight and obese children are less active compared to those with normal weight [7]. The increase in inactivity among children is alarming, particularly because it is known that patterns of physical activity continue from childhood into adulthood [8, 9]. Adolescence is a critical period for the onset of overweight, obesity and obesity-associated morbidity later in life, and 50% to 80% of obese teenagers become obese as adults [10–12]. It is also a period of emotional maturation, with heightened risk for development of anxiety and depression. Being overweight or obese increases this risk [13]. Regular physical activity (PA) decreases many of the negative health consequences of overweight or obesity. It is highly beneficial for both physical and mental health, and is considered to play a key role in treatment and prevention [14]. Several studies on interventions to increase PA level among overweight and obese children have been conducted, but with moderate success and high dropout rates [15]. Newer research suggests that internet technology may be a promising way to help adolescents change their health behaviors [16–20].

In Norway, a 12-week web-based intervention program, called Young & Active, was developed and tested in a controlled trial during the years 2010-2013. The aim was to motivate adolescents with overweight and obesity to increase and maintain physical activity, and thereby enhancing their fitness and health related quality of life. The target group was adolescents aged 13-14, with gender- and age-adjusted BMI above 25. A total of 84 adolescents participated in the intervention group and 36 in the control group. Emphasis in the study was on self-determined PA and not on weight-reduction. The web-based program contained descriptions of what kinds of PA they could register, from organized sports to everyday physical activities, like walking or biking to school, and domestic work. The participants came from 29 different urban and rural schools in south-eastern Norway. Following screening of height and weight in the eighth grade [21], adolescents with age- and gender-adjusted BMI above 25 were invited to take part in the study. The school nurse was involved in the recruitment process, responsible for issuing information and obtaining informed consent from the adolescents and their parents. Self-report instruments, a standardized shuttle-run test, and measurement of height and weight were completed individually three times; at baseline, after the 12 week intervention, and 12 months from baseline. In brief, the 12-week web-based Young & Active intervention included establishing plans and goals for PA, daily registration of PA with continuous graphical feedback on progress, a PA diary, social support in a forum, updated information on PA and nutrition, and weekly, individualized online feedback from the researcher responsible for the quantitative part of the study [22]. The intervention is more thoroughly described elsewhere [23–25]. The Young & Active intervention was built on Self-Determination Theory (SDT) and Motivational Interviewing (MI). SDT is a theory of behavioral motivation, which has proven particularly useful in understanding motivational processes in the context of PA research [26]. According to SDT, people’s ability to engage in and maintain behavior, e.g. physical activity, depends on psychological as well as social-environmental factors. It suggests that all individuals have three key psychological needs: autonomy, competence and relatedness. Support of these needs may promote relatively self-determined forms of motivation for behavioral adaption and maintenance [26], or what SDT characterizes as autonomous motivation. On a continuum, autonomous motivation contrasts with controlled motivation. The latter involves doing an activity as means to an end, and is typically associated with extrinsic rewards, while autonomous motivation involves behaviors performed for their own intrinsic rewards, such as enjoyment or challenge [27]. MI is a collaborative conversation, with the aim of strengthening a person’s own motivation to change by expressing empathy, supporting self-efficacy and increasing awareness on discrepancies between goals and actions [28]. SDT offers a theoretical rationale for understanding how a supportive counseling style like MI can promote health behavior change [29]. The weekly online counseling was based on principles from SDT and MI. Autonomy support was given by exploring the adolescents’ own reasons for change, and showing interest in their well-being. Attention was on how they could make self-determined choices and goals to increase all kinds of PA throughout the day.

As a part of the Young & Active research design, post intervention individual qualitative interviews were conducted with a selection of adolescents from the intervention group. The purpose of the interviews was to get an in-depth understanding of the adolescents’ experiences and perceptions on physical activity, quality of life, and of participation in a web-based intervention.

In this paper, our aim is to describe findings about PA. Specifically, we explore main enablers and barriers which might relate to increasing PA among adolescents with overweight or obesity.

## Methods

We applied a qualitative approach with semi-structured in-depth interviews. Intervention data from the MI counseling sessions and diary notes were used in developing the interview guide. The guide contained questions about the adolescents’ understandings of, and experiences with PA. Other main themes were about their everyday lives at home and in school, their relationships with friends and family, their thoughts about health, and their experiences with participation in Young & Active. For more detailed information about the interview guide, see Appendix. To avoid influencing the results in the quantitative part of the study, the qualitative interviews were conducted immediately after the intervention, and then 9 months later, following the testing schedule in Young & Active. We chose to do two interviews in order to look for both short-term and long-term changes. There were no dropouts between the first and second interviews.

### Recruitment

As to recruitment for the interviews, the first author of this article was present during some of the final testing after the 12-week intervention. This allowed opportunities to inform and to ask the adolescents directly if they were willing to be interviewed. The other participants from the intervention group were informed and asked by the researchers in the Young & Active study. Twenty-one participants, six boys and 15 girls, were recruited from the 84 participants in the intervention, following the same gender distribution as in the main study. The mean age of the adolescents was 13.5 years during the first interview and 14 years during the second interview. A few of the participants belonged to the same school, but most of them came from different schools and districts as in Young & Active. Recruitment proceeded until 21 participants had been interviewed once and data saturation was reached, defined as no new themes emerging, and no further recruitment was necessary [30].

### Data collection

Data was collected and analyzed following the seven phases recommended by Kvale and Brinkmann [31] with thematization, scheduling, interviewing, transcribing, analyzing, verifying, and reporting. The research interview was designed and based on a semi-structured interview guide, seeing interviews as conversations, using main themes as a starting point, followed by open-ended questions, clarifications and probing. The main questions were developed with input from the research group during the process, and discussed until final approval. Before the first interview sessions started, three pilot interviews were conducted to test the interview guide. No changes were found necessary.

### Procedure

The first author conducted two interviews with each participant, a total of 42 interviews. The first interview took place between March and June 2013 and the second interview between March and August 2014, following the progression in the main study. Interviews were conducted during the adolescents’ school hours. The school setting was chosen because it is more naturalistic and familiar for the adolescents. The first interview with each participant was conducted within a few days of completing the Young & Active intervention, due to feasibility. The second interview was held approximately 9 months later. A nine-month timespan was chosen to see if there were changes in their views between the first and the second interviews. The interview sessions started with brief information about the basic aims of the study. Each interview lasted on average 20 min. During the first interview, an agreement on the next interview was made. The first interview was reviewed prior to the second interview, and questions were slightly altered to follow up the adolescents’ answers from the first interview.

### Analysis

All of the 42 interviews were audio-recorded and transcribed verbatim by the first author. Other expressions such as laughter, crying, yawning, sighing, and pauses were noted in brackets. After each interview, additional hand-written field notes about the surroundings, moods, and interruptions were taken, in order to ensure the interpretation of the data. Data were analyzed using qualitative content analysis of the interview text in accordance with recommendations from Graneheim and Lundman [32]. This method was chosen because it allowed us to play an active, reflective role in the interpretation of the participants’ accounts. The text was condensed through a process of abstraction on a higher logical level with emphasis on descriptions and interpretations, and creation of codes, categories and themes [32]. To obtain a sense of the whole, it was listened to and read through several times by the first author. In addition, the co-authors listened to the audio-recordings and read four interviews each, to ensure a common understanding of the meaning and the main themes in the interviews. Main themes were discussed and agreed upon by the group. Transcripts from the first interview were kept separate from the second interview, analyzed independently, compared and finally seen as a whole. Furthermore, they were divided into meaning units, consisting of words and sentences related to each other through their content, condensed meaning units and codes [32]. The final codes from the whole material were compared and sorted into three main themes with several sub-themes. To prevent preconceptions, preliminary themes were discussed between the authors to obtain different perspectives, interpretations and opinions on the text [31]. Based on these discussions, codes and themes were reorganized until consensus was reached. Quotations used in the text have been translated from Norwegian into English.

## Results

Analysis uncovered three main themes: physical activity is synonymous with organized sports and training, physical activity is important for health, and physical activity is fun if one masters the activity (Table 1). Within these themes, there were several sub-themes (Table 1). The themes that we identified were selected in order to explain and understand the motivational processes underpinning physical activity adherence, both facilitating and restraining factors, commonalities and differences. Consistency was found between the first and second interviews.

**Table 1:** Main themes and sub-themes

PA is synonymous with organized sports and exercise	PA is important for health	PA is fun if one masters the activity
• PA is strenuous training • PA means competition • PA is difficult to conduct because of lack of time • PA is not fun when the weather is bad	• PA is important if one wants to have a long life • One must exercise to become thin • PA is necessary to increase fitness and get bigger muscles • Everyone needs to relax	• PA is fun when one is good at it • PA is fun when experiencing a feeling of being in the moment • PA is fun when being together with friends

### Physical activity is synonymous with organized sports and exercise

Interviews revealed that all the participants, when asked about PA, spontaneously started to talk about various kinds of organized sports or exercise, and not about informal active living, like bicycling and walking to school instead of taking the bus or being driven by car, or other informal activities. They did mentioned such activities, but in connection with other questions about their everyday life and not when specifically asked about PA. Even when asked about other activities, several of the adolescents turned the conversation into talk about various kinds of organized sports. Some of the adolescents talked about sports they already took part in, others mentioned PA they would like to have done. One girl said for example: *I’m mostly satisfied with the activities that I participate in but I would really have liked to start volleyball training, but we don’t have that here.* There were minor differences between the boys and the girls in what kinds of sports they mentioned. The boys seemed to be more preoccupied with sports that could help them build bigger muscles or improve fitness. Several of them made comments similar to a boy who said: *Physical activity is fun and it gets you bigger muscles.* The girls talked more about participation in different team sports, and many of them highlighted the social aspects in similar ways to this girl: *It’s easier to exercise together with others; playing handball is very social.* Further underpinning the adolescents’ perceptions of PA as something mainly conducted within organized sports was their association between PA, competition and intense exercise. PA was described as similar to strenuous training; hard at the time, but making one feel good afterwards.

Based on their views of PA as being virtually synonymous with organized sports, other factors like lack of time and weather conditions were mentioned by several interviewees as obstacles to sufficient PA. Even among those who participated in sports regularly, bad weather, like rain or snow, could sometimes affect their motivation to follow up on activities. All of the adolescents described days filled with obligations. Getting grades for the first time seemed to make schoolwork more stressful, and quite a few made statements similar to this boy: *I work hard on schoolwork, it’s a lot, and, you know, I want to do as well as possible.* For some, this was a barrier to continuing in organized sports, or to start new physical activities. The adolescents also talked about other activities, like participation in choirs, scouts, bands, the use of social media, and being with friends as a time barrier to pursuing organized sports. A lack of organized activities during holidays was also mentioned by several interviewees as a challenge to maintaining PA throughout the year. For many, this meant no exercise at all, while others tried to continue on their own, by using fitness equipment at home, jumping on a trampoline, dancing in their room or playing different ball games with friends.

### Physical activity is important for health

When asked about PA, most of the informants linked it to health. A majority of the adolescents said that they experienced their fitness and health as poorer than other youths, and expressed worries not only about their current health, but also about their future health. Many had thoughts about why their health was bad, as one girl stated: *My health is bad…I lack vitamins, I tried to take some, but they were too big, so I stopped…and my fitness is miserable, I can’t run 200 m without getting tired.* Several expressed their perceptions about the connection between PA and health in similar ways to this girl: *Physical activity is very important if one wants to maintain health and have a long life.* Having poor health was associated by the majority with being too heavy. Being heavy made it difficult to perform as well as their fellow peers and some said that this affected their motivation to be physically active or to participate in sports, especially sports demanding stamina like jogging, football or handball. All had perceptions about the importance of physical activity for maintaining health, feeling good and being satisfied with oneself, to quote this boy: *Being healthy, that you are supposed to eat healthy food and exercise, and to have good health, and then you have sort of good fitness…and are sporty.* Both boys and girls said that they thought PA was essential to becoming slimmer, get a more attractive body and improve their health and fitness. For one boy, the wish to become slimmer resulted in a paradoxical effect. He was quite sure that the exercise he did was partly responsible for his increased weight, which made him stop. Several of the adolescents also talked about the importance of relaxation for health. When they had done what they experienced as strenuous training, feeling exhausted, with aching muscles, they felt that they could have a good conscience about relaxing. For some, this feeling seemed to serve as a motivational factor to push themselves to actually do the training. Those who expressed an inability to push themselves also mentioned the importance of relaxation, but rather as an excuse for not being sufficiently physically active, as expressed by this girl: *Everyone needs to relax.*

### Physical activity is fun if one masters the activity

Mastering a physical activity was closely associated with the participants’ experiences of having fun. Several had similar statements to this girl: *Em, I like handball because I think it’s fun, and it’s something that I’m good at, and I have done it for a long time.* When they felt that they were good at an activity, it was easier to just enjoy, relax and have fun, as expressed by this boy:

*Eh, when I ski, then I feel, I begin to be so good that I in a way, I don’t need to concentrate so much, it’s not difficult anymore, I’m just being in the moment, so I think in a way just about that, and not on everything else, schoolwork that I must remember and things like that, I can just relax and be together with friends.*

For some, this feeling of being in the moment wasn’t something that came immediately during PA. They said that they had to push themselves past a point of resistance before they reached this state. Others, who said that the only PA they liked were easy activities that didn’t require physical strain, seemed never to have experienced PA in such a way. They described disliking pushing themselves past the discomfort that PA may give, like one of the girls stated: *It’s a pity, but I can’t say that I like hard physical activities, I think, oh no, not again…and when we finally stop I’m quite ready to do so.* The adolescents’ views about PA also differed as to whether the activities were self-chosen or not. Those who said that they participated in organized sports or training just because their parents or others wanted them to, seemed to perceive PA as less fun and more burdening than those who participated in self-chosen PA.

Growing demands of competition within a sport, with divisions into A and B teams, ending up on the B team, sitting on the bench instead of taking part in the game, were mentioned by some as reasons for quitting the sport. This promoted a feeling of not being good enough. Further, sitting on the bench made the activity boring instead of fun. Some of the adolescents said that they had tried to take up new sports, but decided to quit because they found their teammates to be so much more able. Not knowing the teammates made it even more challenging to be a new team member. Physical education at school was also viewed by a few as too competitive and with too much focus on performance. A couple of girls described episodes where they had been laughed at by their classmates for not mastering an activity. Others mentioned that they thought it was more difficult for them to get good grades in physical education, due to their excess weight and lack of ability to perform according to the criteria.

Friendship meant a lot to the adolescents in this study and was emphasized by all. Being together with friends during sports was largely related to their view of the activity as fun. Outside of school, most of them met up with friends during organized sports or other organized activities. Several made statements similar to this girl:

*Being together with friends on an ordinary day makes me feel better, because then I feel that I have done something, and I have gotten something out of the day. If not, the day has been much more boring, and I’ve just been at home, and on my computer and done things like that.*

Girls who participated in team sports highlighted the team spirit, the opportunity to meet and talk with friends, and the importance of knowing the others. Being together with friends that they had known for a long time made them feel safe, and not afraid of making a fool of themselves. The boys highlighted the ability to compete and talk with their mates during training sessions as important for having fun. One girl, who didn’t attend organized sports anymore because her parents couldn’t afford it, said that she didn’t like the competitive element in sports. She preferred to be together with friends without having to compete.

## Discussion

One aim in the Young & Active intervention was to motivate the adolescents to increase and maintain self-chosen PA of all kinds. We therefore expected to find changes in their perceptions of PA towards an increased awareness about everyday physical activities. Instead, we found that they mainly associated PA with organized sports, health and competence. Viewing PA as synonymous with organized sports may be a significant restraining factor, regardless of the adolescents’ degree or type of motivation to become more physically active. Similar views have been shown in other studies [33, 34]. In modern societies, PA has gone from being necessary and embedded in daily life, to becoming an area organized within different PA disciplines, with standardization of rules, external conditions and measurements of performance. It is adults who define aims, goals and organization, and not the participating children [35]. Participation in PA outside primary school is seldom free of charge, but requires membership fees and often expensive equipment. These changes may have led to a loss of the values of daily, informal active living [36], and may explain why participants in our study associated PA with something conducted within organized sports. For some of the adolescents, sports became difficult to accomplish because of socio-economic and environmental constraints, such as lack of access to localities, lack of equipment and PA options, or high membership fees that their parents couldn’t afford to pay. The seasonal character of organized sports with no training during holidays, bad weather conditions or lack of time also influenced their ability and motivation to do sports on a regular basis. Similar restraining factors are found in other studies [37–41]. In schools, PA is separated into physical education classes. Originally, physical education at school was developed as an alternative to competitive sports, with the aim of promoting students’ development according to their own abilities and skills. However, as within organized sports, this is also governed by rules, expectations and grades, defined by adults [35, 42–44]. When PA is divided into formal versus informal aspects of daily active living, socio-economic, environmental and individual factors may have significant influences on participation in organized PA, and represent important barriers [42].

Among those who already took part in different team sports, increased demands on individual skills seemed to affect their motivation to continue. Being divided into A and B teams made the activity less fun, with a loss of motivation. Others expressed fear of not being sufficiently physically fit or skilled enough to take up new sports because of such expectations. Some described experiences of being stigmatized and laughed at by others due to their inability to perform to a particular standard. Studies show that being overweight or obese increases the risk of social isolation, stigmatization and bullying, and that overweight and obese children are a particularly vulnerable group [42–44].

The majority of the adolescents in this study expressed worries about their health and weight. PA was viewed as essential for obtaining more attractive bodies and improving future health and fitness, both for the girls and the boys. Other studies show similar findings [44, 45]. This may be due to the biomedical constructions of what are considered as acceptable and healthy bodies in our society, seeing overweight or obese bodies as deviant and needing attention to become healthier [46]. Being measured by the school nurse and found to be too heavy may serve to reinforce this impression. A considerable focus on diet, exercise and beauty trends within social media probably also contributes to overweight and obese adolescents’ views on the importance of being physically active to maintain health and to get more attractive bodies [45]. Research shows that adolescents who depart from social norms of attractiveness or individual ideals are at greater risk of developing body dissatisfaction and low self-esteem [13, 44]. Defining real PA as something conducted within organized sports, with requirements many are unable to fulfill, may contribute to such development, and also to a decrease in motivation to maintain or increase PA. This may account for why those of our informants who didn’t succeed looked for explanations, such as being too lazy, too heavy, not having enough time because of other activities, or highlighted the importance of relaxation.

Several of the adolescents in this study expressed positive experiences of participation in organized PA. Having attended team sports for many years, mastering the activity and knowing the other teammates seemed to promote what they described as a state of being in the moment that made the activity fun and not too demanding. Those who described this condition seemed to be significantly more motivated than those who didn’t, and even more so when the sport activity was self-chosen. In terms of STD, these are basic aspects of autonomous motivation [47]. In a similar vein, one study shows that confidence in the ability to be physically active is a positive correlate and a determinant of PA in adolescents [48]. In our study, those who tried to avoid pushing themselves did not describe similar experiences of participation in PA. The reason why some of them still tried to do sports was mostly instrumental, either in order to gain a reward, like losing weight and attaining better health, or to avoid disapproval from teachers or parents, which according to SDT are controlling forms of motivation [47]. Taking up new sports and not knowing the other participants seemed to increase the risk of not liking the activity, and of dropout. Friendship and the opportunity to be together with friends in sports was also an important part of the participants’ perceptions of having fun. It was mentioned by the majority as something that motivated them, both within organized PA, and when taking part in more informal PA activities. All the adolescents in this study reported having friends, though for some, friendship outside of school was difficult to maintain, due to factors such as distance or family situation. These youths reported less time with friends than those who took part in sports. The meaning of friendship and fun for participation in PA is confirmed in several studies. Among overweight adolescents, having fun was shown to be one of the key motivators [43, 49–51]. Other studies show that adolescents who report a greater presence of friends engage more in PA [52]. There is consistent evidence that adolescents with overweight or obesity in general have fewer friends than those who are of normal weight [53–57]. The importance of friendship and engagement in PA seems to be considerable, and a lack of friends may imply less PA among an already vulnerable group. According to SDT, both personal and social-environmental factors facilitate engagement in PA. Studies based on SDT shows that autonomy supportive contexts foster PA [27]. Adolescents in our study, who expressed an inner motivation for participation in organized sports, seemed to be part of more autonomy supportive environments, with fulfilment of basic psychological needs. Those who lacked inner motivation expressed less support, had fewer friends, and felt less competent, which may be considered as barriers to engagement in PA. In terms of SDT, they showed more controlling forms of motivation [27] .

As with all studies, the present study has limitations. The participants belonged to a selected group from the Young & Active intervention, and were probably already preoccupied with PA. To avoid initial influence on their views regarding PA and participation in the web-based program, the interviews were conducted after the intervention. This foreclosed the possibility to compare the adolescents’ pre- and post-intervention views. Some of the youths who were asked to be interviewed declined, and we don’t know their opinions. There is always a risk that participants report views that they think are expected, rather than their true understanding. Nonetheless, the adolescents seemed to generously share their experiences. Important themes arose that may be helpful in understanding some of the complex mechanisms behind PA and inactivity. Several of the findings are supported by other studies.

## Conclusions

The aim of this study was to investigate understandings and experiences of PA among participants from the Young & Active intervention and to identify key factors that foster or undermine motivation for PA. Our results underline the importance of viewing PA in a broader perspective. Culture shapes understandings of PA, body image and health, and PA behavior is a result of interactions between individuals and the multiple contexts in which they are situated. PA was not seen as a natural and necessary part of everyday active living, but as something conducted within organized sports, and as a means of promoting good health and attractive bodies in accordance with social norms of attractiveness. The adolescents’ views on PA as organized sports make it important to look into how these are organized, structured and led. If PA is conducted with pedagogical insight and competence, it may support adolescents’ self-esteem and motivation [35]. Motivation was also related to the adolescents’ experiences of mastering an activity, having fun and being together with friends. The ability to choose among available, affordable and likeable PA, together with friends, may promote participation and maintenance. Incorporating knowledge of overweight or obese adolescents’ understandings and experiences into practice may improve PA interventions. Given our findings, further research on overweight and obese adolescents who have not participated in intervention studies could add important additional knowledge.

## Appendix

Semi-structured interview guide.

1. Family and everyday life

- Whom do you live together with?
- What does a typical day look like for you?
- What do you like to do in your spare time, and together with whom?
- Meal patterns at home, and in school?

2. Friends

- Do you have friends?
- How often do you meet with friends, and what do you do together?

3. School

- How do you experience school (courses, teachers, classmates)?
- Do you participate in physical education classes?

4. Self-image

- What do you think about yourself?
- What do you think others think about you?

5. Mood

- How would you describe your mood?
- What is important for you to feel satisfied in your everyday life?

6. Health

- What are your thoughts about health?
- How do you experience your own health?

7. Sleep

- Do you sleep well?
- What is good sleep for you?

8. Activities

- What kind of activities do you enjoy doing?
- Is there anything that -prevents you from participating in those activities?

9. Physical activity

- Could you tell me your thoughts about physical activity?
- What motivates you to be physically active?
- Is there anything that hinders you from being physically active?

10. Organized sports

- Do you participate in any organized sports? How often?
- How do you experience participation?
- Do you participate for your own enjoyment?

11. Experiences with participation in the research project

- What made you join the Young & Active intervention?
- Was anyone other than you involved in the decision?
- How did you experience the intervention?

The main themes were the same during the second interview, but questions were slightly altered to accommodate the time span.

## Abbreviations

MI: Motivational Interviewing
PA: Physical activity
SDT: Self-determination Theory
