# Correction to: Overweight adolescents’ views on physical activity – experiences of participants in an internet-based intervention: a qualitative study

**DOI:** 10.1186/s12889-018-5546-y

**Published:** 2018-05-14

**Authors:** Turid Kristin Bigum Sundar, Knut Løndal, Per Lagerløv, Kari Glavin, Sølvi Helseth

**Affiliations:** 1 Department of General Practice, Institute of Health and Society, Faculty of Medicine, University of Oslo, Oslo, Norway; 2 Department of Nursing and Health Promotion, Faculty of Health Sciences, OsloMet - Oslo Metropolitan University, Oslo, Norway; 3 Department of Primary and Secondary Teacher Education, Faculty of Education and International Studies, OsloMet -Oslo Metropolitan University, Oslo, Norway; 4 VID Specialized University, Oslo, Norway

## Correction to: BMC Public Health (2018) 18: 448. 10.1186/s12889-018-5324-x

In the original version of this article [1], published on 4 April 2018, there was 1 incorrect author family name. The redundant affiliation (5) has also been removed. The original article has been updated.

The incorrect author name was published as:

- Kari Galvin

The correct author name are:

- Kari Glavin

The original publication of this article has been corrected.
